# Increased risk of nonalcoholic fatty liver disease in patients with thyroid cancer: a nationwide cohort study

**DOI:** 10.1186/s12885-025-14485-2

**Published:** 2025-07-01

**Authors:** Young Bin Cho, Kyoung Sik Park

**Affiliations:** 1 Department of Medicine, Graduate School of Konkuk University, Seoul, 05029 Republic of Korea; 2 Department of Surgery, Konkuk University Medical Center, Seoul, 05029 Republic of Korea; 3 Department of Surgery, Konkuk University School of Medicine, Seoul, 05029 Republic of Korea

**Keywords:** Thyroid cancer, Nonalcoholic fatty liver disease, TSH suppression

## Abstract

**Background:**

Given that patients with thyroid cancer experience several metabolic disorders, it is inferred that these patients have an elevated risk of non-alcoholic fatty liver disease (NAFLD). However, large-scale studies on this topic are lacking. This study aimed to elucidate the association between thyroid cancer and NAFLD.

**Methods:**

We used data from the Korean National Health Insurance Service Sample Cohort. A total of 1,407 patients with thyroid cancer and 4,221 matched controls were included following the exclusion process and propensity score matching at a ratio of 1:3. We analyzed hazard ratio, risk factors for NAFLD.

**Results:**

The study revealed an increased risk of NAFLD (HR 2.28; 95% CI 1.69–3.10; *P* < 0.001) in patients with thyroid cancer. Thyroid cancer patients exhibited a higher risk of NAFLD when they had no regular exercise (HR 2.41; 95% CI 1.75–3.32). Moreover, body mass index (BMI) and levothyroxine dosage have been identified as potential critical factors for the development of NAFLD in patients with thyroid cancer.

**Conclusion:**

The incidence rate of NAFLD was higher in patients with thyroid cancer than in the controls. Healthcare professionals should consider regular physical activity, BMI, and cumulative levothyroxine dosage when managing patients with thyroid cancer to mitigate the incidence of NAFLD.

**Supplementary Information:**

The online version contains supplementary material available at 10.1186/s12885-025-14485-2.

## Introduction

Thyroid cancer is the most common endocrine malignancy, with an increasing incidence over the past few decades [1]. The standard treatment for thyroid cancer, which comprises thyroidectomy and thyroid-stimulating hormone (TSH) suppression therapy, can potentially induce thyroid hormone imbalances, leading to various complications such as metabolic syndrome, cardiovascular abnormalities, and osteoporosis [2]. Given the high survival rates and typically younger age of patients with thyroid cancer, an effective approach is essential for patients to mitigate complications and enhance overall quality of life [3].

Nonalcoholic fatty liver disease (NAFLD) is one of the most prevalent chronic liver conditions worldwide, affecting approximately 38% of the global population [4]. NAFLD poses a substantial socioeconomic burden and is a predominant source of chronic liver disorders. NAFLD encompasses a spectrum of hepatic abnormalities, ranging from steatosis to hepatic fibrosis, cirrhosis, and hepatocellular carcinoma [5, 6].

Thyroid dysfunction is increasingly being recognized as a significant risk factor for NAFLD. A meta-analysis revealed a negative nonlinear correlation between thyroid hormone thyroxine (T4) levels and NAFLD (*p* = 0.003), as well as a U-shaped relationship between TSH levels and the relative risk of NAFLD [7]. Furthermore, Borges-Canha et al. suggested a positive association between TSH levels and the fatty liver index in obese patients [8].

Levothyroxine, employed as TSH suppression therapy for thyroid cancer, is a synthetic analog of T4. It exhibits physiological activity identical to that of T4, which is naturally secreted by the thyroid gland and functions by being converted into triiodothyronine (T3) in peripheral tissues. T3 augments the expression of genes associated with both fatty acid synthesis and β-oxidation, thereby contributing to the dynamic regulation of hepatic lipid metabolism [9]. Reduced levels of T3 or T4 are associated with diminished activity of hepatic lipases, resulting in an increased accumulation of triglycerides. This condition may impair the suppression of lipolysis in adipose tissue, leading to an increased influx of free fatty acids into the liver. Consequently, this can induce hepatic insulin resistance or the development of fatty liver [10].

However, the relationship between thyroid cancer and NAFLD remains uncertain despite previous studies suggesting a potential connection. This uncertainty stems from the fact that the current research has predominantly examined the association between NAFLD and hypothyroidism, with a particular emphasis on subclinical cases. Moreover, large-scale studies exploring the relationship between thyroid cancer and NAFLD are scarce.

In this nationwide cohort study, we aimed to assess the risk of NAFLD between patients with thyroid cancer and individuals without any cancer. To comprehensively explore the relationship between thyroid cancer and NAFLD, we conducted stratified analyses based on age, sex, regular exercise, alcohol binge, smoking status, and comorbidities. Furthermore, we investigated the potential correlation between NAFLD and TSH suppression therapy or body mass index (BMI) in patients with thyroid cancer to explore effective management strategies.

## Materials and methods

### Study population

This study used data from the Korea National Health Insurance Service-Sample Cohort, which included 1,134,108 individual information for qualification, health examination results, medical diagnoses (based on International Classification of Disease, Tenth Revision [ICD-10]), and prescriptions from January 1, 2002, to December 31, 2019.

**Fig. 1:**
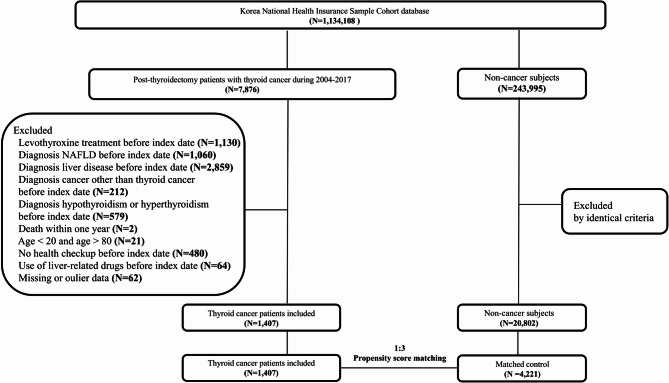
Flow chart of the study population. NAFLD, Nonalcoholic fatty liver disease

We selected prevalent patients who underwent thyroidectomy following a diagnosis of thyroid cancer (C73) between January 1, 2004 and December 31, 2017. The control group comprised individuals who had no diagnosis of malignancies (C00-97, except C73) from January 1, 2002, to December 31, 2019. Subsequently, we applied the following exclusion criteria to both the thyroid cancer and control groups (Fig. 1, Supplementary Table 1): (1) previous history of levothyroxine medication; (2) previous history of NAFLD; (3) diagnosis of liver disease before index date; (4) diagnosis of other malignancies before index date; (5) diagnosis of thyroid disease including hypothyroidism, hyperthyroidism; (6) Death within 1 year after index date; (7) age under 20 years or age over 80 years; (8) absence of health checkup before index date; (9) Use of drugs which could be associated with incidence of liver disease before index date; (10) Missig or outlier data [11]. In the cohort data, the presence of an outlier may indicate a potential issue with the patient’s record, thereby rendering the reliability of other variable values questionable. Therefore, we excluded all outlier data which constituted about 3% of the total data to prevent potential bias in the findings (as detailed in Appendix A).

For thyroid cancer patients, the index date was determined as either the initial levothyroxine prescription date for those receiving treatment or the first thyroidectomy date for those not receiving levothyroxine. The index date for the control group was the date of the first health examination. Events occurring after a 5-year period from the index date were censored to assess the risk rate within the initial 5 years. We applied 5-year censoring to standardize follow-up duration across participants and to minimize potential bias due to differential follow-up time.

### Outcomes and measures

The primary outcome was the incidence of NAFLD (K76.0). The follow-up period was defined as the time interval between the index date and the date of the study outcome, death, or end of the study period (December 31, 2019).

Patients with thyroid cancer were further categorized according to the thyroidectomy type (total thyroidectomy or lobectomy) and levothyroxine administration. The average daily dose per weight of levothyroxine (µg/day/kg) was calculated as the cumulative levothyroxine dose divided by the total number of prescription days up to the time of the event and body weight.

### Covariates

Qualification and health examination data were used to assess demographic characteristics at the index date. Residential areas were categorized into two categories: urban (Seoul, Busan, Daegu, Incheon, Gwangju, Daejeon, and Ulsan) and rural (Gyeonggi Province, Gangwon Province, North and South Chungcheong Provinces, North and South Jeolla). Income was stratified into three levels: lower 40%, middle 30%, and upper 30% [12]. Smoking status was classified as never, ex-, or current.

Alcohol intake was defined as the frequency of weekly alcohol consumption. Alcohol binge was defined according to criteria published in the National Health and Nutrition Survey. For participants enrolled between 2004 and 2008, consuming more than one bottle of Korean liquor was considered an alcohol binge.; after 2009 alcohol binge was defined as more than 5 drinks per session in male or 4 drinks per session in female, respectively [13]. Regular exercise was defined as exercise at least 3 times per week [14]; Dyslipidemia was defined as the use of the ICD-10 code E78 or total cholesterol level of ≥ 240 mg/dL. Diabetes was defined as the use of ICD-10 code E10-14 or fasting plasma glucose (FPG) ≥ 126 mg/dL. Hypertension was defined as the use of ICD-10 codes I10-13 or I15 or blood pressure ≥ 140/90 mmHg [15]. Obesity was defined as a BMI ≥ 25 kg/m^2^ [16].

### Statistical analysis

Descriptive statistics were used to characterize the baseline characteristics. Continuous variables are presented as mean and standard deviation. Categorical variables were presented as absolute numbers and percentages. The Student T- test, based on normality, was used to compare continuous variables between the two groups, whereas the chi-squared test was used to assess categorical variables. Using propensity score matching (PSM), we created well-matched groups while minimizing the impact of confounding factors. In this study, the statistical method of PSM was employed to match individuals between the two groups in a 1:3 ratio. Logistic regression estimates the propensity score model, wherein each subject’s estimated probability of being in the thyroid group, based on the given covariates, corresponds to the propensity score. We used age, sex, BMI, residential area, disability, regular exercise, systolic blood pressure (SBP), diastolic blood pressure (DBP), FPG, alanine aminotransferase (ALT), aspartate aminotransferase (AST), gamma-glutamyl transferase (GGT), and total cholesterol as potential confounders. Caliper was set at 0.02 and greedy nearest neighbor matching was used. The covariate balance between the thyroid cancer and control groups was assessed using the standardized mean difference (SMD). An SMD value of less than 0.2 was deemed to indicate negligible imbalance. A love plot was used to visualize the distribution of SMD across covariates and to demonstrate improvement in covariate balance after PSM.

Cox proportional hazards regression was used to calculate the hazard ratio of NAFLD between the thyroid cancer and control groups. The cumulative incidence of NAFLD was evaluated using a Kaplan-Meier curve, and differences between the two groups were assessed using the log-rank test. Multivariate analyses were performed using the Cox proportional hazards regression model, which was adjusted for age, sex, BMI, residential area, income level, disability, smoking status, alcohol intake, alcohol binge, regular exercise, SBP, DBP, FPG, AST, ALT, GGT, total cholesterol, dyslipidemia, diabetes, and hypertension. Stratification analyses were visualized using a forest plot. We characterized the association of NAFLD with the cumulative dosage of levothyroxine, levothyroxine daily dosage per weight, or BMI, which was treated as a continuous variable. We used a restricted cubic spline analysis, with five knots located at the 5th, 25th, 50th, 75th, and 95th percentiles of the cumulative dosage of levothyroxine, levothyroxine daily dosage per weight, or BMI. The reference values were 180 mg for cumulative levothyroxine dosage, 1.56 µg/day/kg for daily levothyroxine dosage per weight, and 23 kg/m^2^ for BMI.

Data preprocessing was performed using the SAS software (version 9.4; SAS Institute Inc., Cary, NC, USA). Statistical analyses and visualizations were conducted using the R software version 4.0.3 (R Project, Vienna, Austria). Two-sided *P* values of < 0.05 were considered statistically significant.

## Results

### Characteristics of the thyroid cancer patients and controls

The study initially comprised 1,407 individuals diagnosed with thyroid cancer and 20,802 control subjects before the application of PSM (Supplementary Table 2). Following PSM at a 1:3 ratio, the final cohort consisted of 1,407 patients with thyroid cancer and 4,221 matched controls (Table 1). The mean age of participants was mid-40s, with females representing 78% of the study population, and the average body mass index (BMI) was approximately 23 kg/m^2^. The follow-up durations were 4.87 years of thyroid cancer group and 4.48 years of the control group. To visually present covariate balance before and after PSM, a love plot was presented in Supplementary Fig. 1.

**Table 1:** Baseline characteristics of the study population

	Thyroid cancer (*N* = 1,407)	Control (*N* = 4,221)	SMD
Age, years	46.92 (10.88)	45.44 (11.28)	0.134
Sex			< 0.001
Male	306 (21.7)	918 (21.7)	
Female	1101 (78.3)	3,303 (78.3)	
BMI, kg/m^2^	23.68 (3.35)	23.09 (4.41)	0.150
Residential area			0.027
Urban	713 (50.7)	2,197 (52.0)	
Rural	694 (49.3)	2,024 (48.0)	
Income level			0.425
Low	361 (25.7)	1,780 (42.2)	
Middle	374 (26.6)	1,213 (28.7)	
High	672 (47.8)	1,228 (29.1)	
Disability	37 (2.6)	58 (1.4)	0.090
Smoking status			0.218
Never	1,184 (84.2)	3,417 (81.0)	
Ex	103 (7.3)	188 (4.5)	
Current	120 (8.5)	616 (14.6)	
Alcohol intake			0.204
< 1	991 (70.4)	3,305 (78.3)	
1–2	324 (23.0)	668 (15.8)	
3–4	76 (5.4)	174 (4.1)	
≥ 5	16 (1.1)	74 (1.8)	
Alcohol binge	486 (34.5)	3,102 (73.5)	0.849
Regular exercise	171 (12.2)	615 (14.6)	0.071
SBP, mmHg	120.12 (15.24)	121.95 (17.23)	0.112
DBP, mmHg	75.32 (10.35)	76.18 (11.14)	0.079
Lab measurements			
FPG, mg/dL	94.58 (17.14)	93.15 (21.68)	0.073
ALT, U/L	21.30 (14.10)	20.55 (20.09)	0.044
AST, U/L	22.13 (9.14)	22.99 (12.75)	0.077
GGT, U/L	23.08 (18.57)	25.32 (29.82)	0.090
Total cholesterol, mg/dL	194.70 (35.96)	194.40 (36.07)	0.008
Comorbidities			
Dyslipidemia	477 (33.9)	476 (11.3)	0.562
Diabetes	205 (14.6)	185 (4.4)	0.353
Hypertension	460 (32.7)	876 (20.8)	0.272
Obesity	515 (36.6)	878 (20.8)	0.355

Values are presented as mean (standard deviation) for continuous variables and number (%) for categorical variables

*BMI* Body mass index, *SBP* Systolic blood pressure, *DBP* Diastolic blood pressure, *FPG* Fasting plasma glucose, *ALT* Alanine aminotransferase, *AST* Aspartate aminotransferase, *GGT* Gamma-glutamyl transferase, *SMD* Standardized matching difference

Among patients with thyroid cancer, 1,061 individuals (75%) underwent total thyroidectomy, as detailed in Supplementary Table 3. Additionally, 1,319 patients (93%) received levothyroxine therapy, as indicated in Supplementary Table 4. Notable differences were observed between the groups receiving levothyroxine and those not receiving treatment. Specifically, the mean BMI was significantly higher in the levothyroxine group (23.73 kg/m^2^) than in the group not receiving levothyroxine (22.88 kg/m^2^), with a statistical significance of *P* = 0.021. Furthermore, the prevalence of obesity was significantly higher in the levothyroxine group than in the non-levothyroxine group (37% vs. 25%, *P* = 0.026).

### Increased risk of NAFLD in patients with thyroid cancer compared to controls

**Fig. 2:**
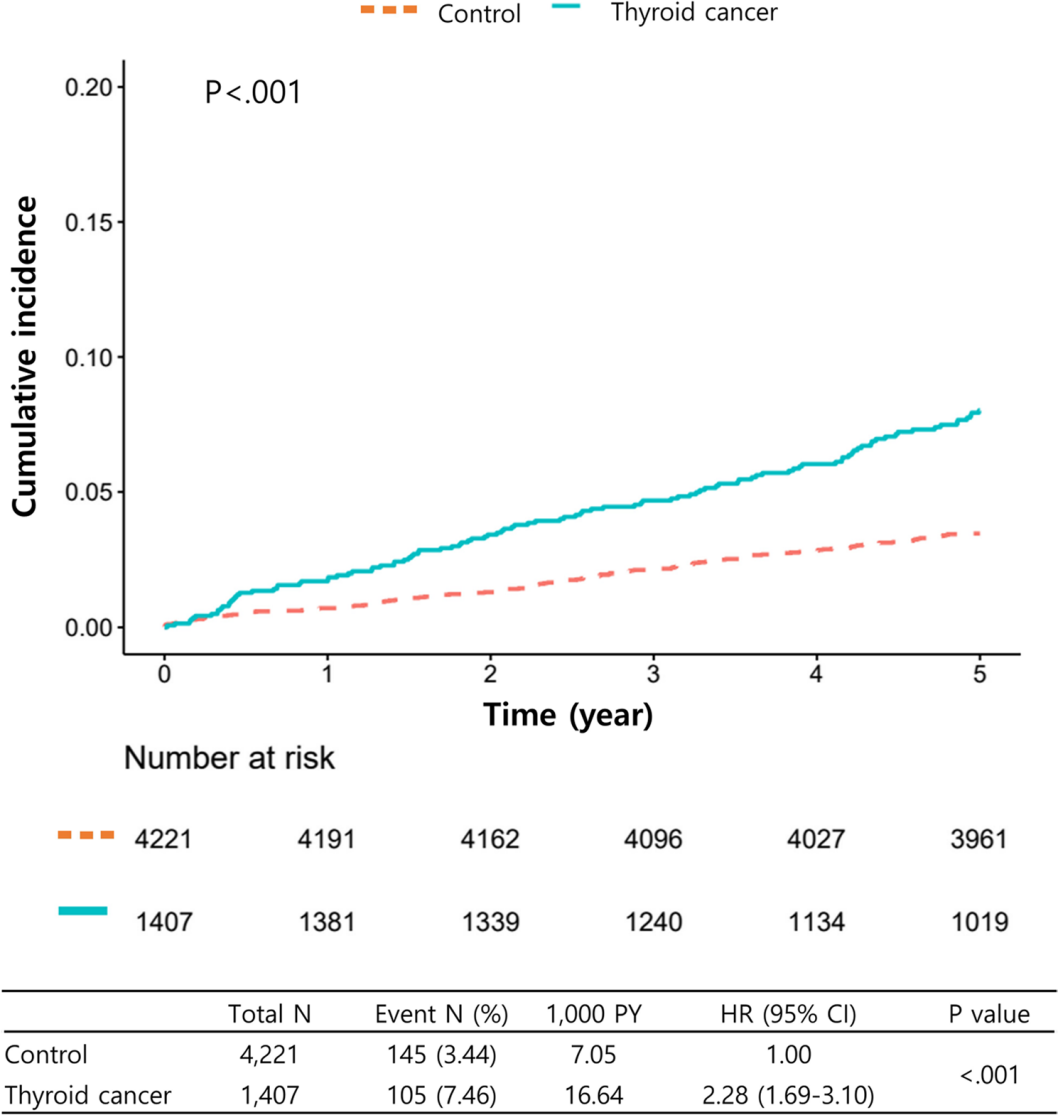
Cumulative incidence of NAFLD in thyroid cancer group and matched control group. Kaplan–Meier curve presents the incidence rate of NAFLD in the total population. NAFLD, nonalcoholic fatty liver disease; PY, person-year; HR, hazard ratio; CI, confidence interval

**Fig. 3:**
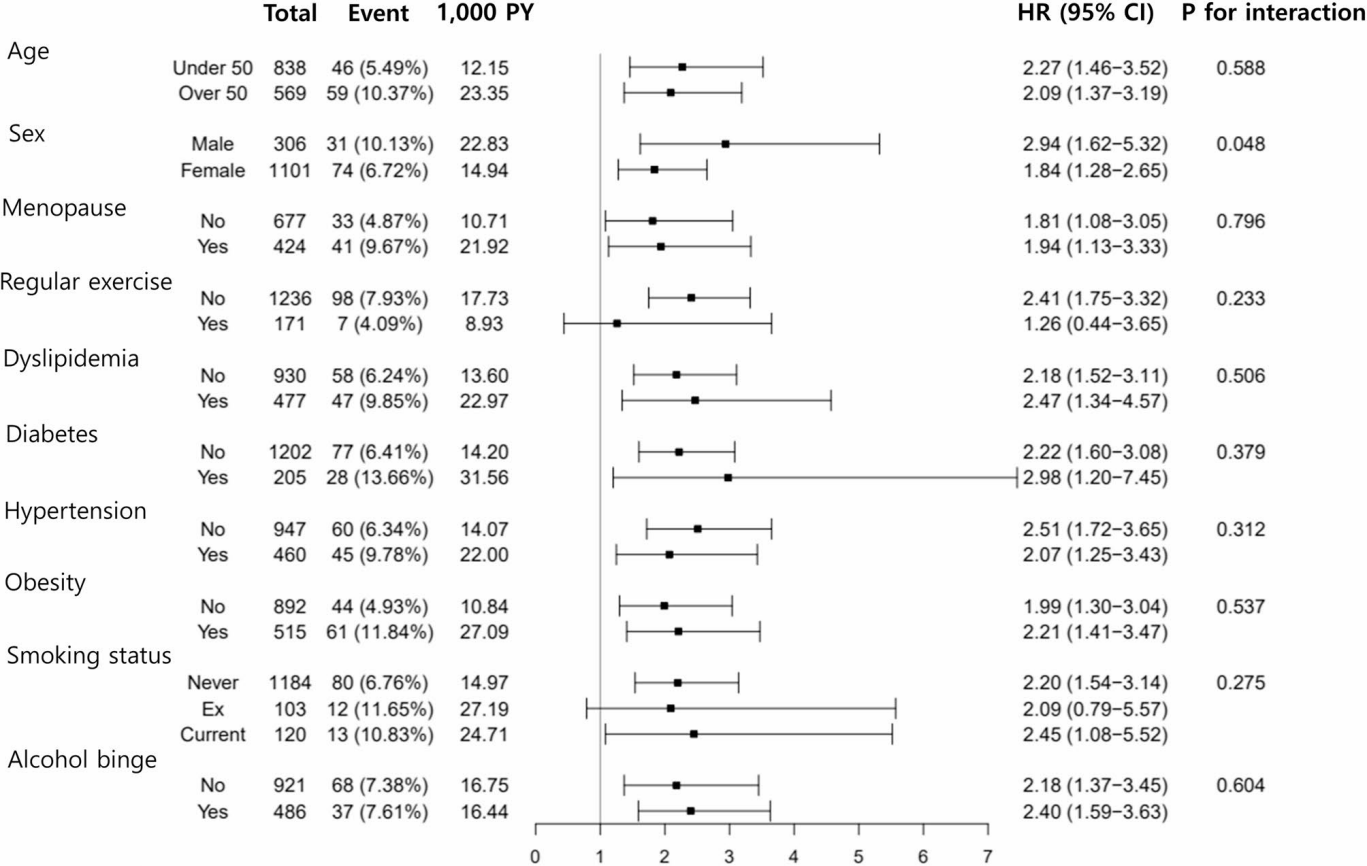
Stratification analyses of NAFLD in thyroid cancer group compared to control. Forest plot presents the stratification analysis. NAFLD, nonalcoholic fatty liver disease; PY, person-year; HR, hazard ratio; CI, confidence interval

Figure 2 presented the risk estimates of NAFLD between the thyroid cancer group and the control group. The thyroid cancer group exhibited a significantly higher incidence of NAFLD than the control (hazard ratio [HR] 2.28; 95% confidence interval [CI] 1.69–3.10; *P* < 0.001). Figure 3 depicted the stratification analysis in the form of a forest plot. Female participants were stratified into two cohorts based on their age at the index date: those under 50 years (no menopause) and those 50 years or older (menopause). Regardless of subgroups, such as age, sex, menopause, comorbidities, and alcohol consumption, the thyroid cancer group demonstrated a significantly elevated risk of NAFLD compared to the control.

Thyroid cancer patients who did not engage in regular exercise at the index date showed a considerably elevated risk of NAFLD compared to the control group (HR 2.41; 95% CI 1.75–3.32). Conversely, no significant difference was observed between the patients and controls who engaged in regular exercise on the index date. Furthermore, never-smokers and current smokers exhibited a significantly higher risk of NAFLD in the thyroid cancer group than in the control group. However, among the ex-smokers, the incidence of NAFLD did not differ significantly between the thyroid cancer and control groups.

Cox regression and stratification analyses before PSM were presented in Supplementary Fig. 2 and Supplementary Fig. 3. In addition, stratification analyses by comorbidities were given in Supplementary Tables 5 and Supplementary Table 6.

### Effect of thyroidectomy, levothyroxine, BMI on NAFLD in patients with thyroid cancer

Table 2 presented the risk of NAFLD in thyroid cancer patients, considering factors such as thyroidectomy and levothyroxine treatment. The analysis revealed no statistically significant differences based on either the type of thyroidectomy or levothyroxine administration. However, when the levothyroxine dose was stratified into subgroups, the risk of developing NAFLD was significantly elevated in individuals receiving ≤ 120 mg levothyroxine compared to those not receiving levothyroxine, while administration of > 180 mg was associated with a significant decrease in risk of NAFLD.

**Table 2:** Risk of NAFLD among thyroid cancer patients

	Total	Event (%)	1,000 PY	HR (95% CI)	*P* value
Thyroidectomy					
Lobectomy	346	24 (6.94%)	17.07	1.00	
Total thyroidectomy	1,061	81 (7.63%)	16.51	0.88 (0.55–1.39)	0.581
Levothyroxine treatment					
Without treatment	88	8 (9.09%)	21.35	1.00	
With treatment	1,319	97 (7.35%)	16.34	0.64 (0.31–1.34)	0.237
≤ 120 mg	250	61 (24.40%)	78.73	4.24 (1.97–9.11)	< 0.001
120–180 mg	132	18 (13.64%)	32.85	1.56 (0.67–3.65)	0.304
> 180 mg	668	18 (2.69%)	5.51	0.19 (0.08–0.45)	< 0.001

Estimates were adjusted for age, sex, body mass index, residential area, income level, disability, smoking status, alcohol intake, alcohol binge, regular exercise, systolic blood pressure, diastolic blood pressure, fasting plasma glucose, alanine aminotransferase, aspartate aminotransferase, gamma-glutamyl transferase, total cholesterol, dyslipidemia, diabetes, and hypertension

*NAFLD* Nonalcoholic fatty liver disease, *PY* person-year, *HR* Hazard ratio, *CI* Confidence interval

**Fig. 4:**
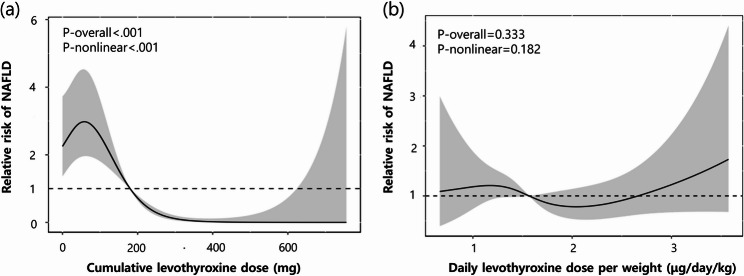
Relative risk of NAFLD in patients with thyroid cancer by levothyroxine dosage. Restricted cubic spline curves characterize the association between levothyroxine dose (X-axis) and risk of NAFLD (Y-axis). (**a**) cumulative levothyroxine dose and (**b**) daily levothyroxine dose per weight. The solid line indicates the relative HR and the shaded are represents the 95% CI. The reference values are (**a**) 180 mg, (**b**) 1.56 µg/day/kg, respectively (HR = 1.0). The spline curve was constructed using 5 knots, specifically placed at (**a**) 0, 127.6, 278.6, 414.7, 589.2 mg, (**b**) 0.9, 1.3, 1.6, 1.9, 2.7 µg/day/kg. NAFLD, nonalcoholic fatty liver disease; HR, hazard ratio; CI, confidence interval

The relative hazard ratio for NAFLD was plotted against levothyroxine dosage using restrictive cubic spline curves. Figure 4a indicated a significant association between the cumulative levothyroxine dose and the relative risk of developing NAFLD in both general and nonlinear statistical correlations. However, no significant correlation was observed between the daily levothyroxine dose per body weight and the relative risk of NAFLD, as shown in Fig. 4b.

**Fig. 5:**
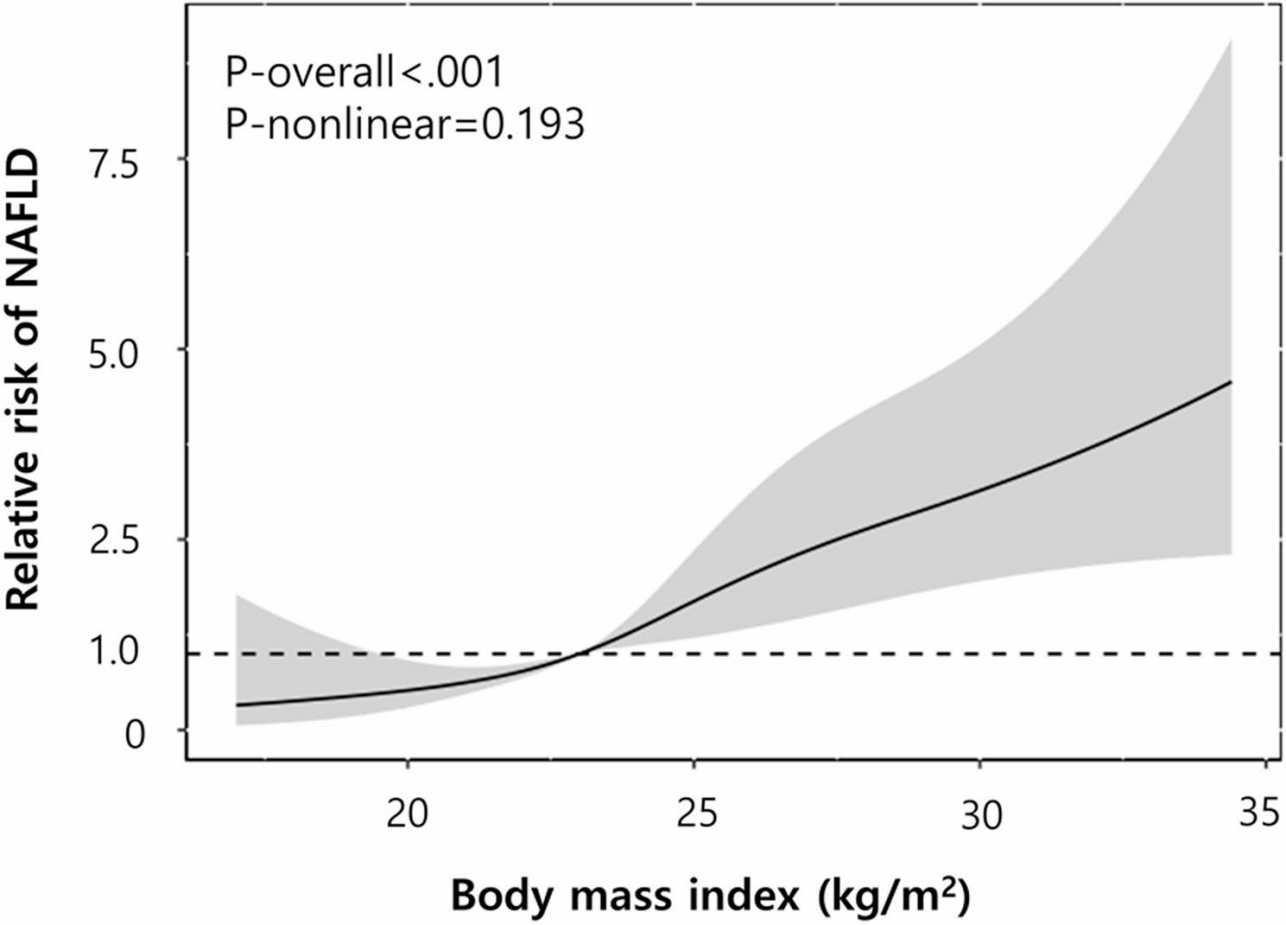
Relative of NAFLD in patients with thyroid cancer by BMI. Restricted cubic spline curve characterizes the association between BMI (X-axis) and risk of NAFLD (Y-axis). The solid line indicates the relative HR and the shaded are represents the 95% CI. The reference value is 23 kg/m^2^ (HR = 1.0). The spline curve was constructed using 5 knots, specifically placed at 18.8, 21.4, 23.4, 25.6, 29.4 kg/m^2^. NAFLD, nonalcoholic fatty liver disease; BMI, body mass index; HR, hazard ratio; CI, confidence interval

Moreover, the relative hazard ratio for NAFLD in relation to BMI was represented as a cubic curve using a restrictive cubic spline (Fig. 5). The analysis demonstrated a statistically significant association between BMI and NAFLD risk (*P* < 0.001), but a nonlinear relationship was not significant (*P* = 0.193).

## Discussion

This study is the first population-based study to demonstrate an increased risk of NAFLD in patients with thyroid cancer, compared to matched controls. Our study indicates that screening for NAFLD is essential in patients with thyroid cancer. Moreover, this may support a bidirectional association between thyroid cancer and NAFLD. Previous research has identified NAFLD as a risk factor for developing thyroid cancer, and even young adults with NAFLD exhibited a higher risk of thyroid cancer [17]. These findings with our study highlight the complex relationship between thyroid cancer and NAFLD and suggest that each condition may influence the development of the other.

Stratification analyses indicated the risk of developing NAFLD in patients with thyroid cancer was significantly higher than that in the controls, when regular exercise was not performed. However, in the regular exercise group, there was no statistically significant difference in the incidence of NAFLD between the two groups. Furthermore, the thyroid cancer group exhibited only seven outcomes, rendering it challenging to derive statistical significance. In addition, the overall risk of developing NAFLD increased significantly as BMI increased in patients with thyroid cancer. It suggests that adjusting the BMI may be associated with a reduced risk of NAFLD in patients with thyroid cancer. This analysis is consistent with previous studies that have identified BMI as a key predictor of fatty liver development [13, 18]. In a long-term retrospective study of Japanese, NAFLD incidence was compared according to BMI quartiles, confirming that higher BMI quartiles were associated with a higher prevalence of NAFLD [18]. Changes were also predictors of NAFLD incidence [13, 18]. A small increase in BMI resulted in a high rate of NAFLD in the higher BMI group [18].

Thyroidectomy can be associated with conditions similar to hypothyroidism in patients with thyroid cancer. Previous studies have demonstrated that hypothyroidism decreases the lipid disposal rate, while the normalization of thyroid hormone levels in patients with hypothyroidism results in a significant increase in the lipid disposal rate [19, 20]. This suggests that hypothyroidism may contribute to lipid abnormalities, including elevations in total cholesterol, low-density lipoprotein cholesterol, and triglycerides, and a reduction in fatty acid oxidation. Several studies have demonstrated that administration of levothyroxine may restore lipid metabolism in patients with hypothyroidism [18, 21, 22]. In a double-blind, placebo-controlled study, hypothyroidism patients with levothyroxine treatment showed improved lipid metabolism after 6 months of stable euthyroid state [21]. However, there is a lack of large-scale, well-designed prospective studies that specifically examine whether thyroid hormone replacement therapy is associated with a decreased risk of NAFLD. Our study revealed that hazard ratio of NAFLD in the levothyroxine treatment group was lower than that in the no-treatment group among patients with thyroid cancer, but the difference was not statistically significant. Additionally, we stratified thyroid cancer patients into three groups according to the cumulative dosage of levothyroxine and found that the risk of developing NAFLD was relatively lower in the high-dose group than in the no-treatment group, whereas there were no significant results in the medium-dose group, and the risk of developing NAFLD was higher in the low-dose group.

We also analyzed the correlation between levothyroxine dosage and NAFLD development using a restricted spline curve. The observed nonlinear relationship between cumulative levothyroxine doses and the relative risk of NAFLD suggests the possibility of a rapid increase in risk beyond a certain dosage threshold, or the existence of a plateau interval. Clinically, it is preferable to avoid cumulative doses exceeding a specific threshold and to enhance monitoring of liver function and fatty liver in individuals receiving high doses. Besides, the result suggests that levothyroxine dosage might be more closely associated with the cumulative dose level than the daily dosage per weight to prevent NAFLD. Furthermore, the analysis of the restricted cubic spline curve concerning BMI reveals that an increase in BMI is significantly associated with an elevated risk of NAFLD, consistent with established research [23, 24]. However, the lack of statistical significance in the nonlinear relationship implies a consistent increase in NAFLD risk with higher BMI, without a point of rapid risk escalation beyond a certain BMI level. Consequently, BMI can be an independent risk factor for NAFLD, with particular emphasis on liver disease risk management in obese patients.

This study had some limitations. First, the database contains no information on histology of thyroid cancer, including papillary, medullary, follicular, and anaplastic thyroid carcinoma. Further investigation is required to determine whether these histological subtypes of thyroid cancer have an impact on the osteoporosis risk. Additionally, the database lacks information on thyroid function tests, such as TSH, T4, and T3 levels. Although levothyroxine dosage is frequently employed as an indirect measure of TSH suppression, this limitation may have the possibility of misclassification, particularly in patients with altered absorption, metabolic rate of thyroid hormones. Future research should employ laboratory-based cohorts that include comprehensive thyroid function test results to more accurately quantify the extent of TSH suppression and its association with NAFLD. Second, although this study revealed a correlation between NAFLD incidence and BMI or cumulative levothyroxine dosage, it did not determine specific cut-off values for these parameters. Establishing such thresholds would be beneficial for developing more precise clinical guidelines. Third, due to the observational design of this study, causal inferences cannot be drawn. Although adjustments were made for a variety of potential confounding variables, these findings should be interpreted as associations rather than causal effects. Further prospective and experimental studies are necessary to validate these results. Lastly, SMD for certain variables, such as alcohol binge, remained elevated following PSM. To address this imbalance, we adjusted for all confounding factors to analyze the hazard ratio of NAFLD.

Nevertheless, this study has several strengths. First, it included a large nationwide cohort of the Korean population, which may reflect the characteristics of the entire population and may include a long-term follow-up period. Second, the study employed strict exclusion criteria and adjusted for potential confounders, rendering it less susceptible to bias compared to case-control studies. Third, it elucidates factors for monitoring the risk of NAFLD through stratification analysis and examination of cumulative levothyroxine dosage.

This study is large-scale cohort study about the risk of NAFLD in patients with thyroid cancer. This study offers valuable evidence to support early identification and intervention strategies for metabolic complications. The statistically significant predictors identified herein may serve as essential feature sets for future machine learning-and deep learning–based risk prediction models. Recent research has demonstrated the efficacy of optimization-based AI approaches in medical prediction tasks. For instance, the integration of Greylag Goose Optimization (GGO) and Multilayer Perceptron (MLP) has enhanced the diagnostic accuracy for lung cancer [25], while Binary Particle Swarm Optimization (BPSO)has improved the performance of COVID-19 predictive models [26, 27]. Additionally, the application of metaheuristic-based algorithms such as Modified Al-Biruni Earth Radius (MBER) has resulted in significant advancements in electroencephalography-based classification tasks [28], and a Convolutional Neural Network - Long Short-Term Memory (CNN-LSTM) has achieved 97.1% accuracy in the early detection of potato diseases by capturing both spatial and temporal features from complex input data [29]. Aligned with these advancements, the findings of this study can be extended to form a foundation for developing high-accuracy AI-enhanced NAFLD prediction systems utilizing hybrid deep learning architectures optimized by metaheuristic algorithms. This could ultimately facilitate the implementation of intelligent risk stratification tools within medical record systems or digital healthcare platforms for patients with thyroid cancer.

## Conclusions

In conclusion, this study revealed that thyroid cancer might be associated with the risk of developing NAFLD compared with non-cancer participants. In addition, BMI and levothyroxine dosage could be critical factors in the development of NAFLD in thyroid cancer patients. To reduce the incidence of NAFLD, medical experts should consider BMI, and cumulative levothyroxine dosage when treating patients with thyroid cancer.

## Supplementary Information

Supplementary Material 1: Supplementary Figure 1. Love plot of SMD before and after PSM. A love plot presents covariates balance between thyroid cancer group and control group before (blue circles) and after (red circles) PSM. The vertical dotted line indicates SMD = 0.1. SMD, standardized mean differences; GGT, gamma-glutamyl transferase;; ALT, Alanine aminotransferase; AST, aspartate aminotransferase; SBP, systolic blood pressure; DBP, diastolic blood pressure; BMI, body mass index; FPG, fasting plasma glucose.

Supplementary Material 2: Supplementary Figure 2. Before PSM, cumulative incidence of NAFLD in thyroid cancer group and matched control group. Kaplan–Meier curve presents the incidence rate of NAFLD in the total population. NAFLD, nonalcoholic fatty liver disease; PY, person-year; HR, hazard ratio; CI, confidence interval.

Supplementary Material 3: Supplementary Figure 3. Before PSM, incidence of NAFLD in thyroid cancer group and matched control group. Forest plot presents the stratification analysis. NAFLD, nonalcoholic fatty liver disease; PY, person-year; HR, hazard ratio; CI, confidence interval.

Supplementary Material 4.

Supplementary Material 5.

## Data Availability

Due to the National Health Insurance policy restrictions, the original datasets cannot be exported or shared outside the institution. All data generated or analyzed during this study are included in this published article. For further inquiries, please contact the corresponding author.

## Abbreviations

NAFLD: Non-alcoholic fatty liver disease
BMI: Body mass index
TSH: Thyroid-stimulating hormone
T4: Thyroxine
T3: Triiodothyronine
SBP: Systolic blood pressure
DBP: Diastolic blood pressure
FPG: Fasting plasma glucose
ALT: Alanine aminotransferase
AST: Aspartate aminotransferase
GGT: Gamma-glutamyl transferase
PSM: Propensity score matching
SMD: Standard mean difference
HR: Hazard ratio
CI: Confidence interval
