# Supplementary figures and images for: Increased risk of nonalcoholic fatty liver disease in patients with thyroid cancer: a nationwide cohort study
Source: BMC Cancer. 2025 Jul 1;25:1093. doi: 10.1186/s12885-025-14485-2 (PMC12210664; doi:10.1186/s12885-025-14485-2)

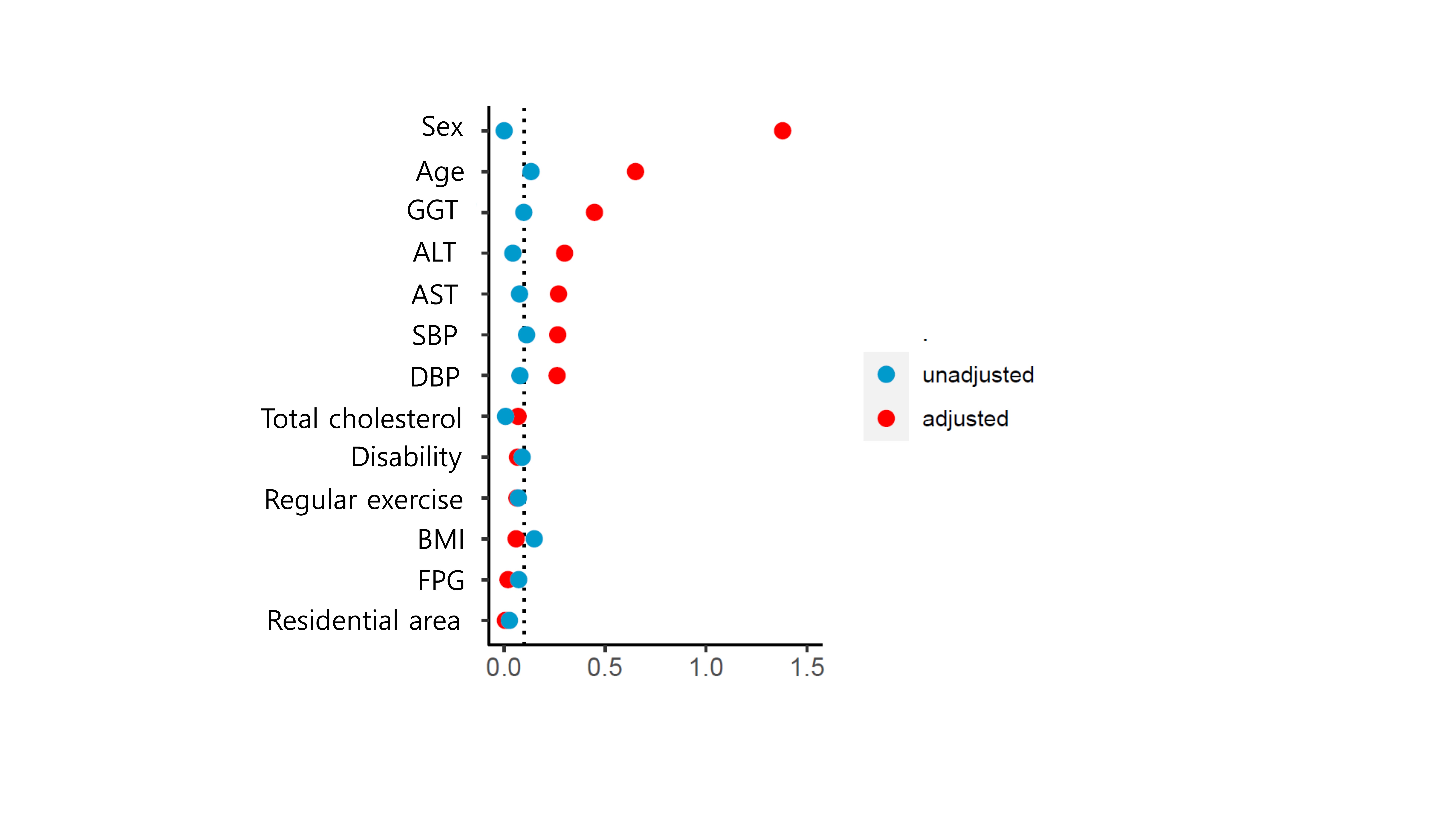

Supplement: Supplementary file 1 — Supplementary Material 1: Supplementary Figure 1. Love plot of SMD before and after PSM. A love plot presents covariates balance between thyroid cancer group and control group before (blue circles) and after (red circles) PSM. The vertical dotted line indicates SMD = 0.1. SMD, standardized mean differences; GGT, gamma-glutamyl transferase;; ALT, Alanine aminotransferase; AST, aspartate aminotransferase; SBP, systolic blood pressure; DBP, diastolic blood pressure; BMI, body mass index; FPG, fasting plasma glucose. [file 12885_2025_14485_MOESM1_ESM.jpg]

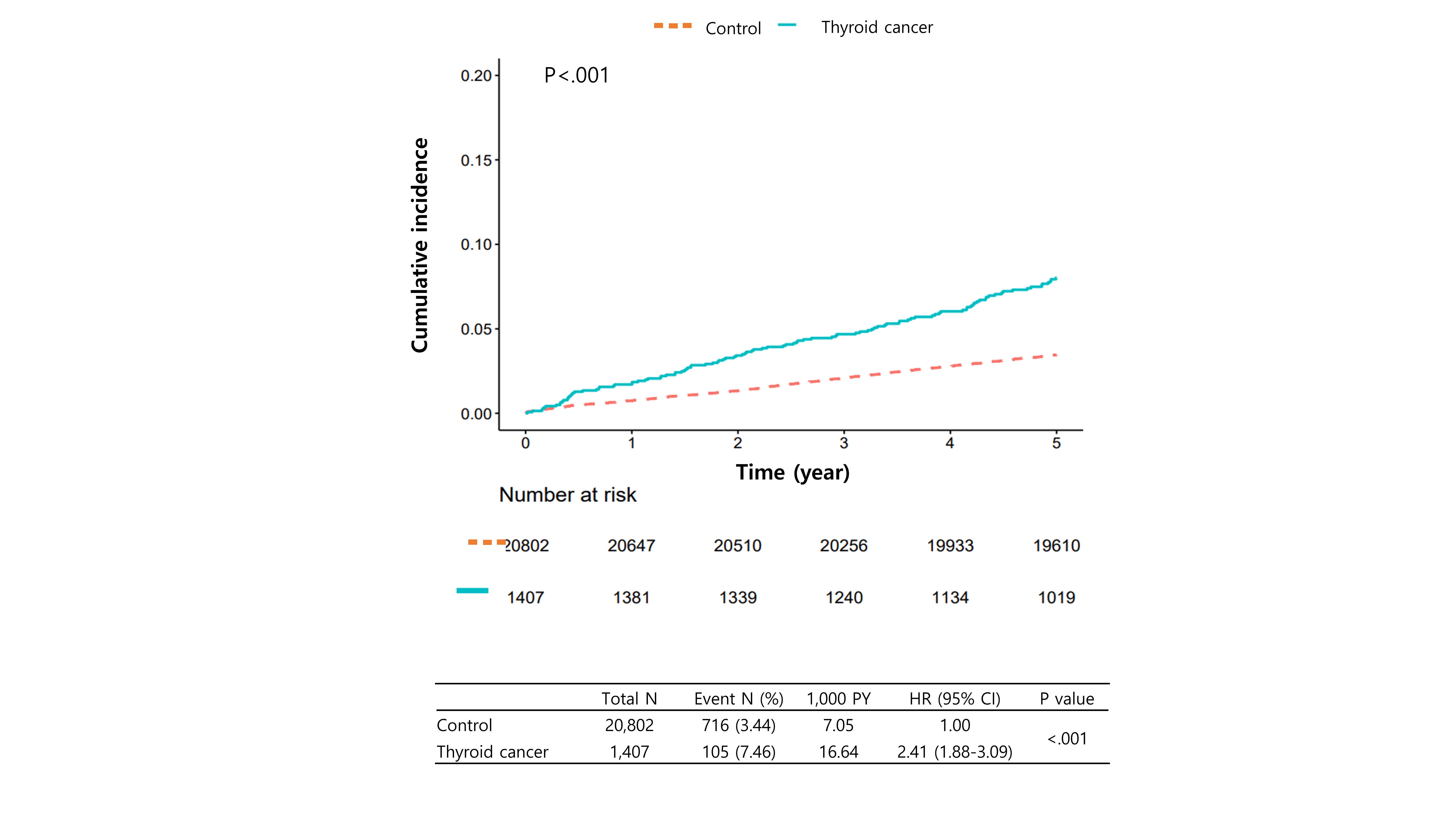

Supplement: Supplementary file 2 — Supplementary Material 2: Supplementary Figure 2. Before PSM, cumulative incidence of NAFLD in thyroid cancer group and matched control group. Kaplan–Meier curve presents the incidence rate of NAFLD in the total population. NAFLD, nonalcoholic fatty liver disease; PY, person-year; HR, hazard ratio; CI, confidence interval. [file 12885_2025_14485_MOESM2_ESM.jpg]

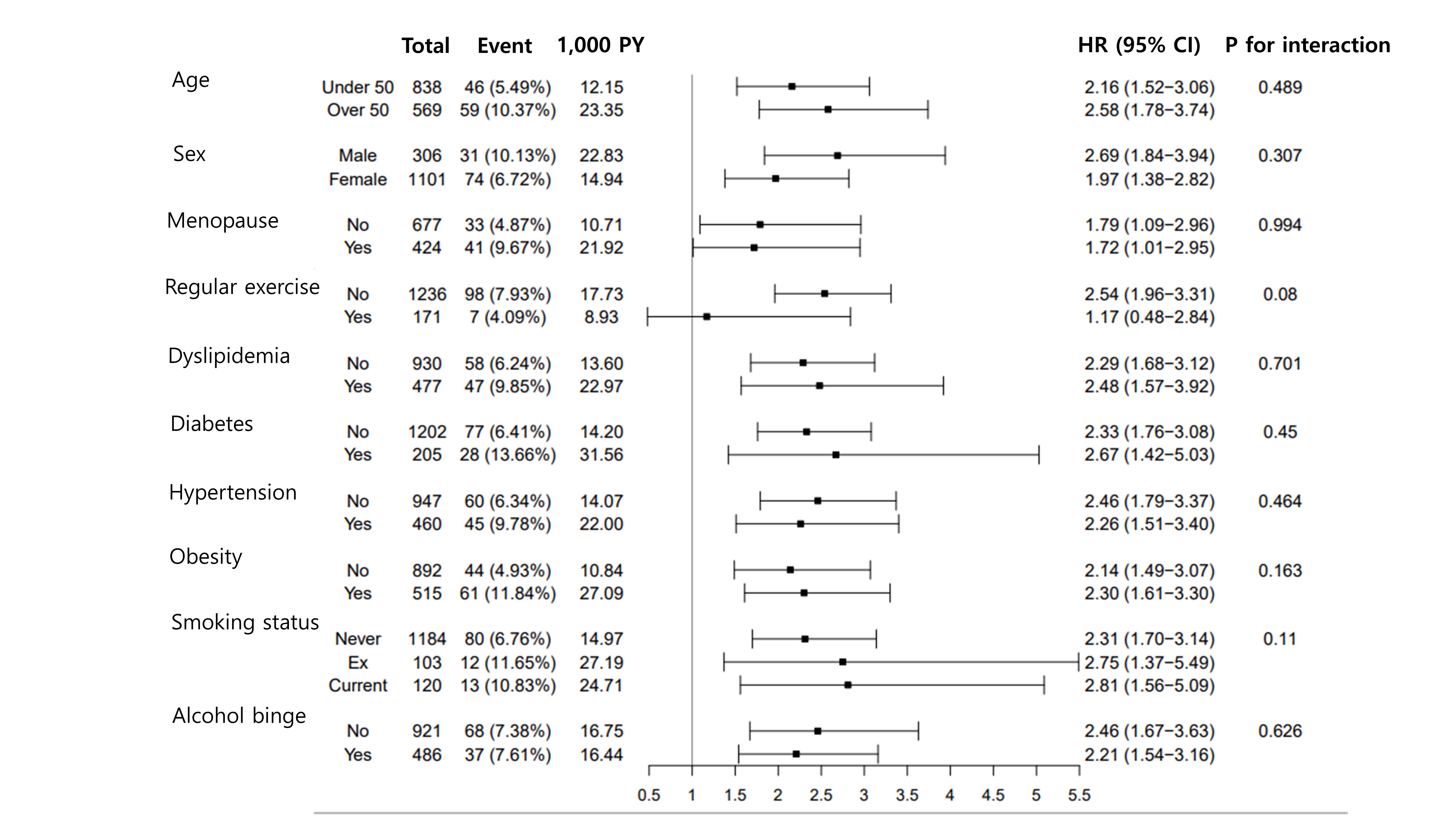

Supplement: Supplementary file 3 — Supplementary Material 3: Supplementary Figure 3. Before PSM, incidence of NAFLD in thyroid cancer group and matched control group. Forest plot presents the stratification analysis. NAFLD, nonalcoholic fatty liver disease; PY, person-year; HR, hazard ratio; CI, confidence interval. [file 12885_2025_14485_MOESM3_ESM.jpg]
