# Supplementary material for: Increased risk of nonalcoholic fatty liver disease in patients with thyroid cancer: a nationwide cohort study
Source: BMC Cancer. 2025 Jul 1;25:1093. doi: 10.1186/s12885-025-14485-2 (PMC12210664; doi:10.1186/s12885-025-14485-2)
Supplement: Supplementary file 4 — Supplementary Material 4. [file 12885_2025_14485_MOESM4_ESM.docx]

Supplementary Table 1. Definitions of outcome, comorbidities, exposure, and exclusion criteria.

|  | **Type** | **Codes** |
| --- | --- | --- |
| ***Outcome*** | | |
| NAFLD | ICD-10 | K76.0 |
| ***Comorbidities*** | | |
| Dyslipidemia | ICD-10 | E78 |
| Diabetes | ICD-10 | E10, E11, E12, E13, E14, E15 |
| Hypertension | ICD-10 | I10, I11, I12, I13, I15 |
| ***Exposure*** |  |  |
| Levothyroxine | ATC | 183601ATB,183602ATB,183603ATB,183604ATB,  183606ATB,183607ATB, 183608ATB,183609ATB,183610ATB,183611ATB |
| Total thyroidectomy | KHIPC | P4561, P4552 |
| Lobectomy | KHIPC | P4551, P4553, P4554 |
| ***Exclusion*** |  |  |
| Other cancer | ICD-10 | C00-C96 except C73 |
| Hypothyroidism | ICD-10 | E03 |
| Hyperthyroidism | ICD-10 | E05 |
| Steroid | ATC | 193302ATB, 193305ATB, 217034ASY, 217001ATB, 193601BIJ, 193603BIJ, 193604BIJ, 217131COS, 217132COS, 217135COS, 217136COS, 217137COS, 217138COS, 217139COS, 193432COM, 193433COM, 193434CLT, 193435CLT, 193438CLT, 217531CCM, 217532CCM, 217533CCM, 217534CCM, 217535CLT, 217536CCM, 217537CCM, 141901ATB, 141903ATB, 142230BIJ,142232BIJ, 141930COO, 141931COS, 141906CIM, 142231COO, 141910CSI, 142130CCM, 142131CCM, 142133CCM, 243201ATB,243202ATB, 243203ATB, 243336BIJ, 243335BIJ, 243337BIJ, 243340COS, 243330CLQ, 243331CCM, 243339COM, 160201ATB, 170901ATB, 170906ATB, 170931COM, 170932CCM, 170947COM, 170938CLT, 170939CLT, 170942CLT, 170943CLT, 170933CSS, 170950CLT, 170941CLT, 170944CLT, 170945CLT, 170946CLT, 170934CSS, 171030CCM, 171130CCM |
| Valproic acid | ATC | 229734ASY, 229701ATR, 229703ATB, 229705ATR, 229706ATR, 229707ATR, 246901ATE, 246902ATE, 246903ATE, 247002ACS, 229730BIJ, 229731BIJ, 229704BIJ |
| Amiodarone | ATC | 107401ATB, 107430BIJ |
| Tamoxifen | ATC | 234501ATB, 234502ATB |
| Methotrexate | ATC | 192101ATB, 192132BIJ, 192134BIJ, 192136BIJ, 192144BIJ, 192142BIJ, 192143BIJ, 192139BIJ, 192141BIJ |
| Anti-convulsant | ATC | 146830BIJ, 509330BIJ, 509331BIJ, 123130ASY, 123102ATB, 123104ATR |
| Proton-pump inhibitor | ATC | 204401ACE, 204401ATE, 204402ATE, 204403ATE, 367201ACH, 367201ACR, 367201ATB, 367202ACH, 367202ACR, 367202ATB, 367203ACR, 367203ATB, 498001ACH, 498002ACH, 509902ACH, 459401BIJ, 367201ATB, 367202ATB, 208801ATE, 208802ATE, 519201ATE, 519202ATE, 656701ATE, 208801BIJ |

NAFLD, Nonalcoholic fatty liver disease; ATC, Anatomical therapeutic chemical classification; ICD-10, International classification of disease 10th revision; KHIPC, Korean Health Insurance Procedure Code.

Supplementary table 2. Baseline characteristics of thyroid cancer group and control group before propensity score matching.

|  | **Thyroid cancer**  **(N=1,407)** | **Control**  **(N=20,802)** | **SMD** |
| --- | --- | --- | --- |
| Age, years | 46.92 (10.88) | 39.59 (11.63) | 0.651 |
| Sex |  |  | 1.379 |
| Male | 306 (21.7) | 16,331 (78.5) |  |
| Female | 1,101 (78.3) | 4,471 (21.5) |  |
| BMI, kg/m^2^ | 23.68 (3.35) | 23.47 (3.53) | 0.060 |
| Residential area |  |  | 0.007 |
| Urban | 713 (50.7) | 10,470 (50.3) |  |
| Rural | 694 (49.3) | 10,332 (49.7) |  |
| Income level |  |  | 0.444 |
| Low | 361 (25.7) | 8,015 (38.5) |  |
| Middle | 374 (26.6) | 7,180 (34.5) |  |
| High | 672 (47.8) | 5,607 (27.0) |  |
| Disability | 37 (2.6) | 345 (1.7) | 0.067 |
| Smoking status |  |  | 0.969 |
| Never | 1,184 (84.2) | 9,247 (44.5) |  |
| Ex | 103 (7.3) | 2,071 (10.0) |  |
| Current | 120 (8.5) | 9,484 (45.6) |  |
| Alcohol intake |  |  | 0.279 |
| <1 | 991 (70.4) | 12,083 (58.1) |  |
| 1-2 | 324 (23.0) | 6,227 (29.9) |  |
| 3-4 | 76 (5.4) | 1,871 (9.0) |  |
| ≥5 | 16 (1.1) | 621 (3.0) |  |
| Alcohol binge | 486 (34.5) | 16,453 (79.1) | 1.007 |
| Regular exercise | 171 (12.2) | 2,977 (14.3) | 0.064 |
| SBP, mmHg | 120.12 (15.24) | 124.26 (15.91) | 0.266 |
| DBP, mmHg | 75.32 (10.35) | 78.13 (10.94) | 0.263 |
| **Lab measurements** |  |  |  |
| FPG, mg/dL | 94.58 (17.14) | 94.14 (25.58) | 0.020 |
| ALT, U/L | 21.30 (14.10) | 27.25 (24.19) | 0.300 |
| AST, U/L | 22.13 (9.14) | 25.62 (15.85) | 0.270 |
| GGT, U/L | 23.08 (18.57) | 39.98 (49.99) | 0.448 |
| Total cholesterol, mg/dL | 194.70 (35.96) | 192.18 (36.42) | 0.070 |
| **Comorbidities** |  |  |  |
| Dyslipidemia | 477 (33.9) | 2,461 (11.8) | 0.545 |
| Diabetes | 205 (14.6) | 1,102 (5.3) | 0.314 |
| Hypertension | 460 (32.7) | 5,468 (26.3) | 0.141 |
| Obesity | 515 (36.6) | 5,452 (26.2) | 0.225 |

Values are presented as mean (standard deviation) for continuous variables and number (%) for categorical variables. BMI, body mass index; SBP, systolic blood pressure; DBP, diastolic blood pressure; FPG, fasting plasma glucose; ALT, Alanine aminotransferase; AST, aspartate aminotransferase; GGT, gamma-glutamyl transferase; SMD, Standardized matching difference.

Supplementary table 3. Baseline characteristics in thyroid cancer patients according to thyroidectomy type.

|  | **Total thyroidectomy**  **(N=1,061)** | **Lobectomy**  **(N=346)** | **P value** |
| --- | --- | --- | --- |
| Age, years | 46.96 (11.04) | 46.80 (10.38) | 0.810 |
| Sex |  |  | 0.736 |
| Male | 228 (21.49) | 78 (22.54) |  |
| Female | 833 (78.51) | 268 (77.46) |  |
| BMI, kg/m^2^ | 23.75 (3.43) | 23.46 (3.09) | 0.134 |
| Residential area |  |  | 0.885 |
| Urban | 536 (50.52) | 177 (51.16) |  |
| Rural | 525 (49.48) | 169 (48.84) |  |
| Income level |  |  | 0.204 |
| Low | 284 (26.77) | 77 (22.25) |  |
| Middle | 282 (26.58) | 92 (26.59) |  |
| High | 495 (46.65) | 177 (51.16) |  |
| Disability | 26 (2.45) | 11 (3.18) | 0.588 |
| Smoking status |  |  | 0.759 |
| Never | 897 (84.54) | 287 (82.95) |  |
| Ex | 75 (7.07) | 28 (8.09) |  |
| Current | 89 (8.39) | 31 (8.96) |  |
| Alcohol intake |  |  | 0.043 |
| <1 | 767 (72.29) | 224 (64.74) |  |
| 1-2 | 228 (21.49) | 96 (27.75) |  |
| 3-4 | 53 (5.00) | 23 (6.65) |  |
| ≥ 5 | 13 (1.23) | 3 (0.87) |  |
| Alcohol binge | 376 (35.44) | 110 (31.79) | 0.241 |
| Regular exercise | 130 (12.25) | 41 (11.85) | 0.917 |
| SBP, mmHg | 120.54 (15.59) | 118.84 (14.03) | 0.057 |
| DBP, mmHg | 75.55 (10.46) | 74.62 (9.98) | 0.147 |
| **Lab measurements** |  |  |  |
| FPG, mg/dL | 94.64 (17.05) | 94.39 (17.44) | 0.819 |
| ALT, U/L | 21.46 (14.30) | 20.84 (13.47) | 0.48 |
| AST, U/L | 22.13 (9.08) | 22.16 (9.31) | 0.957 |
| GGT, U/L | 22.88 (18.06) | 23.69 (20.06) | 0.504 |
| Total cholesterol, mg/dL | 194.32 (36.70) | 195.86 (33.60) | 0.471 |
| **Comorbidities** |  |  |  |
| Dyslipidemia | 350 (32.99) | 127 (36.71) | 0.229 |
| Diabetes | 154 (14.51) | 51 (14.74) | 0.988 |
| Hypertension | 349 (32.89) | 111 (32.08) | 0.831 |
| Obesity | 391 (36.85) | 124 (35.84) | 0.783 |

Values are presented as mean (standard deviation) for continuous variables and number (%) for categorical variables. BMI, body mass index; SBP, systolic blood pressure; DBP, diastolic blood pressure; FPG, fasting plasma glucose; ALT, Alanine aminotransferase; AST, aspartate aminotransferase; GGT, gamma-glutamyl transferase; SMD, Standardized matching difference.

Supplementary table 4. Baseline characteristics in thyroid cancer patients according to levothyroxine.

|  | **With levothyroxine**  **(N=1,319)** | **Without levothyroxine**  **(N=88)** | **P value** |
| --- | --- | --- | --- |
| Age, years | 46.97 (10.92) | 46.15 (10.29) | 0.490 |
| SEX |  |  | 0.923 |
| Male | 286 (21.68) | 20 (22.73) |  |
| Female | 1,033 (78.32) | 68 (77.27) |  |
| BMI, kg/m^2^ | 23.73 (3.35) | 22.88 (3.18) | 0.021 |
| Residential area |  |  | 0.390 |
| Urban | 664 (50.34) | 49 (55.68) |  |
| Rural | 655 (49.66) | 39 (44.32) |  |
| Income level |  |  | 0.823 |
| Low | 336 (25.47) | 25 (28.41) |  |
| Middle | 351 (26.61) | 23 (26.14) |  |
| High | 632 (47.92) | 40 (45.45) |  |
| Disability | 35 (2.65) | 2 (2.27) | 1.000 |
| Smoking status |  |  | 0.117 |
| Never | 1,116 (84.61) | 68 (77.27) |  |
| Ex | 92 (6.97) | 11 (12.50) |  |
| Current | 111 (8.42) | 9 (10.23) |  |
| Alcohol intake |  |  | 0.189 |
| <1 | 936 (70.96) | 55 (62.50) |  |
| 1-2 | 298 (22.59) | 26 (29.55) |  |
| 3-4 | 69 (5.23) | 7 (7.95) |  |
| ≥ 5 | 16 (1.21) | 0 (0.0) |  |
| Alcohol binge | 458 (34.72) | 28 (31.82) | 0.661 |
| Regular exercise | 163 (12.36) | 8 (9.09) | 0.460 |
| SBP, mmHg | 120.15 (15.33) | 119.70 (13.80) | 0.791 |
| DBP, mmHg | 75.30 (10.33) | 75.65 (10.74) | 0.761 |
| **Lab measurements** |  |  |  |
| FPG, mg/dL | 94.80 (17.00) | 91.22 (18.96) | 0.057 |
| ALT, U/L | 21.38 (14.31) | 20.17 (10.54) | 0.312 |
| AST, U/L | 22.15 (9.27) | 21.88 (6.90) | 0.725 |
| GGT, U/L | 23.09 (18.69) | 23.00 (16.71) | 0.966 |
| Total cholesterol, mg/dL | 194.75 (35.97) | 193.92 (35.91) | 0.834 |
| **Comorbidities** |  |  |  |
| Dyslipidemia | 448 (33.97) | 29 (32.95) | 0.938 |
| Diabetes | 197 (14.94) | 8 (9.09) | 0.177 |
| Hypertension | 429 (32.52) | 31 (35.23) | 0.685 |
| Obesity | 493 (37.38) | 22 (25.00) | 0.026 |

Values are presented as mean (standard deviation) for continuous variables and number (%) for categorical variables. BMI, body mass index; SBP, systolic blood pressure; DBP, diastolic blood pressure; FPG, fasting plasma glucose; ALT, Alanine aminotransferase; AST, aspartate aminotransferase; GGT, gamma-glutamyl transferase; SMD, Standardized matching difference.

Supplementary table 5. Stratification analysis of NAFLD by comorbidities after PSM.

|  |  |  | Total N | Event N (%) | 1,000PY | HR  (95% CI) | P value |
| --- | --- | --- | --- | --- | --- | --- | --- |
| Dyslipidemia | No | Control | 3,745 | 124 (3.31%) | 6.79 | 1.00 |  |
|  |  | TC | 930 | 58 (6.24%) | 13.60 | 2.18  (1.52-3.11) | <.001 |
|  | Yes | Control | 476 | 21 (4.41%) | 9.09 | 1.00 |  |
|  |  | TC | 477 | 47 (9.85%) | 22.97 | 2.47  (1.34-4.57) | 0.004 |
| Diabetes | No | Control | 4,036 | 134 (3.32%) | 6.81 | 1.00 |  |
|  |  | TC | 1,202 | 77 (6.41%) | 14.20 | 2.22  (1.60-3.08) | <.001 |
|  | Yes | Control | 185 | 11 (5.95%) | 12.50 | 1.00 |  |
|  |  | TC | 205 | 28 (13.66%) | 31.56 | 2.98  (1.20-7.45) | 0.019 |
| Hypertension | No | Control | 3,345 | 96 (2.87%) | 5.87 | 1.00 |  |
|  |  | TC | 947 | 60 (6.34%) | 14.07 | 2.51  (1.72-3.65) | <.001 |
|  | Yes | Control | 876 | 49 (5.59%) | 11.65 | 1.00 |  |
|  |  | TC | 460 | 45 (9.78%) | 22.00 | 2.07  (1.25-3.43) | 0.005 |
| Obese | No | Control | 3,343 | 97 (2.90%) | 5.94 | 1.00 |  |
|  |  | TC | 892 | 44 (4.93%) | 10.84 | 1.99  (1.30-3.04) | 0.001 |
|  | Yes | Control | 878 | 48 (5.47%) | 11.34 | 1.00 |  |
|  |  | TC | 515 | 61 (11.84%) | 27.09 | 2.21  (1.41-3.47) | <.001 |

Estimates were adjusted for age, sex, body mass index, residential area, income level, disability, smoking status, alcohol intake, alcohol binge, regular exercise, systolic blood pressure, diastolic blood pressure, fasting plasma glucose, alanine aminotransferase, aspartate aminotransferase, gamma-glutamyl transferase, total cholesterol, dyslipidemia, diabetes, and hypertension. PSM, propensity score matching; PY, person-year; HR, hazard ratio; CI, confidence interval. TC, thyroid cancer group.

Supplementary table 6. Stratification analysis of NAFLD by comorbidities before PSM.

|  |  |  | Total N | Event N (%) | 1,000PY | HR  (95% CI) | P value |
| --- | --- | --- | --- | --- | --- | --- | --- |
| Dyslipidemia | No | Control | 18,341 | 582 (3.17%) | 6.49 | 1.00 |  |
|  |  | TC | 930 | 58 (6.24%) | 13.60 | 2.29  (1.68-3.12) | <.001 |
|  | Yes | Control | 2,461 | 134 (5.44%) | 11.32 | 1.00 |  |
|  |  | TC | 477 | 47 (9.85%) | 22.97 | 2.48  (1.57-3.92) | <.001 |
| Diabetes | No | Control | 19,700 | 638 (3.24%) | 6.62 | 1.00 |  |
|  |  | TC | 1,202 | 77 (6.41%) | 14.20 | 2.33  (1.76-3.08) | <.001 |
|  | Yes | Control | 1,102 | 78 (7.08%) | 14.89 | 1.00 |  |
|  |  | TC | 205 | 28 (13.66%) | 31.56 | 2.67  (1.42-5.03) | 0.002 |
| Hypertension | No | Control | 15,334 | 440 (2.87%) | 5.86 | 1.00 |  |
|  |  | TC | 947 | 60 (6.34%) | 14.07 | 2.46  (1.79-3.37) | <.001 |
|  | Yes | Control | 5,468 | 276 (5.05%) | 10.46 | 1.00 |  |
|  |  | TC | 460 | 45 (9.78%) | 22.00 | 2.26  (1.51-3.40) | <.001 |
| Obese | No | Control | 15,350 | 440 (2.87%) | 5.85 | 1.00 |  |
|  |  | TC | 892 | 44 (4.93%) | 10.84 | 2.14  (1.49-3.07) | <.001 |
|  | Yes | Control | 5,452 | 276 (5.06%) | 10.47 | 1.00 |  |
|  |  | TC | 515 | 61 (11.84%) | 27.09 | 2.30  (1.61-3.30) | <.001 |

Estimates were adjusted for age, sex, body mass index, residential area, income level, disability, smoking status, alcohol intake, alcohol binge, regular exercise, systolic blood pressure, diastolic blood pressure, fasting plasma glucose, alanine aminotransferase, aspartate aminotransferase, gamma-glutamyl transferase, total cholesterol, dyslipidemia, diabetes, and hypertension. PSM, propensity score matching; PY, person-year; HR, hazard ratio; CI, confidence interval. TC, thyroid cancer group.
