# Supplementary material for: Increased risk of nonalcoholic fatty liver disease in patients with thyroid cancer: a nationwide cohort study
Source: BMC Cancer. 2025 Jul 1;25:1093. doi: 10.1186/s12885-025-14485-2 (PMC12210664; doi:10.1186/s12885-025-14485-2)
Supplement: Supplementary file 5 — Supplementary Material 5. [file 12885_2025_14485_MOESM5_ESM.docx]

**Appendix A.**

**Outlier definition criteria**

To ensure data quality and minimize the risk of bias due to implausible or erroneous values, outliers were defined and excluded based on predetermined thresholds for each clinical and laboratory variable. The table below presents the criteria used to identify outlier values.

| **No.** | **Variable** | **Outlier Criteria** |
| --- | --- | --- |
| 1 | Systolic blood pressure (SBP) | < 60 mmHg or > 400 mmHg |
| 2 | Diastolic blood pressure (DBP) | < 30 mmHg or > 250 mmHg |
| 3 | Fasting plasma glucose (FPG) | < 25 mg/dL or > 999 mg/dL |
| 4 | Total cholesterol | < 400 mg/dL or > 999 mg/dL |
| 5 | Aspartate aminotransferase (AST) | > 999 U/L |
| 6 | Alanine aminotransferase (ALT) | > 999 U/L |
| 7 | Gamma-glutamyl transferase (GGT) | > 999 U/L |
